# A Qualitative Analysis of the Barriers to Healthcare and Education for Adolescent Girls in Tanzania

**DOI:** 10.7759/cureus.52384

**Published:** 2024-01-16

**Authors:** Varvara Ermioni Triantafyllidi, Mundhir Semm Ally Basinda, Margaret Sylvester Tayari, Ahlam Amour, Nassor Rashid Hamad, Ferenc Macsali, Lina Michala

**Affiliations:** 1 1st Department of Obstetrics and Gynecology, ‘Alexandra’ General Hospital, National and Kapodistrian University of Athens, Athens, GRC; 2 Obstetrics and Gynecology, Mnazi Mmoja Hospital, Zanzibar, TZA; 3 Obstetrics and Gynecology, Haukeland University Hospital, Bergen, NOR

**Keywords:** africa, tanzania, zanzibar, life quality, qualitative research, education, contraception, family planning, adolescent pregnancy

## Abstract

Introduction: Tanzania has a high adolescent birth rate and many sexually active adolescents do not have access to effective contraception. Teenage pregnancy is considered a high-risk pregnancy. Furthermore, it leads to social inequalities for both mother and offspring.

Methods: We conducted semistructured interviews with 12 adolescent mothers during their stay in the postnatal ward of the maternity department of a tertiary hospital in Zanzibar. The study took place between November and December 2022. Data were then analyzed qualitatively.

Results: The main theme that emerged from the interview data was that pregnancy seemed to affect the lives of young girls in a negative way. The majority of pregnancies were unplanned, and the girls reported low family planning uptake. Another recurring theme was that girls had many οbstacles in their education prior to pregnancy, which left them uncertain about their future. Finally, despite the advice of local doctors, the majority of the girls received minimal prenatal care, mostly because they did not regard obstetric care to be a priority.

Conclusions: Adolescent pregnancy remains an important public health issue in Tanzania, despite significant measures by authorities to reduce it. Educational changes and professional opportunities as well as family planning services may enable young girls to achieve professional and personal goals while delaying motherhood into adulthood.

## Introduction

Tanzania has a high adolescent birth rate; 22% percent of women aged 20 to 24 years have given birth before turning 18 [1-2]. Until recently, adolescent pregnant girls were banned from attending and continuing public secondary education programs, leading to social, financial, and educational discrimination toward them [1]. Although family planning provisions have greatly improved recently, it is estimated that only 34.3% of the population uses some form of contraception and 41% of girls in this age group have an unmet need for modern contraception [3].

In the meantime, teenage births continue to create social inequalities and are considered high-risk pregnancies. There is an increased risk of anemia, preeclampsia, and eclampsia, as well as preterm birth and low birth weight of the neonate [4-5]. Children born by teenage mothers are more likely to have developmental delays and be disadvantaged, socially and financially [4-5].

Therefore, the aim of this project was to assess the perceptions and expectations of adolescent pregnant women regarding the quality and accessibility of the healthcare system and the barriers that they faced, their knowledge and use of contraception methods, as well as their aspirations about their future and the future of their offspring. 

This article was previously presented as an oral presentation at the 20th World Congress of Paediatric and Adolescent Gynaecology (WCPAG), Belgrade, May 2023 and the abstract has been published in the Congress Book of WCPAG, 2023. 

## Materials and methods

This was a qualitative study based on a semi-structured interview, with postpartum adolescent African women based on content analysis (Appendix 1). We interviewed in total 12 adolescent mothers. The number of interviews may be small but since this is a qualitative study we focused on the researcher's close association with the respondents to enhance the validity of fine-grained, in-depth inquiry. 

Participants were recruited from a postnatal clinic in Mnazi Mmoja Hospital (MMH) in Zanzibar. The obstetric department was informed about the project through flyers and a brief introductory presentation. Midwives informed V.E.T. (first author and principal investigator) of any possible participants. The inclusion criteria were African women, age <19 years, and delivery of a healthy newborn. We excluded girls whose pregnancy was the result of sexual assault and girls who suffered from any physical or mental condition. Interviews took place at the postnatal clinic and they lasted between 40 and 90 minutes.

The study received ethical approval from the Zanzibar Health Research Committee (ZAHREC), (Ref: NO.ZAHREC/04/PR/NOV/2022/38) as well as from the scientific board of MMH. Prior to the interview, we obtained written informed consent from each adolescent girl and her guardian parent (Appendix 2).

Semi-structured, in-depth interviews were conducted between November and December of 2022. Prior to the interview, we collected demographic data and the medical and obstetric records of each girl (Appendix 1). The semi-structured interviews focused on the following points: (1) the feelings of the adolescent girl towards their recent pregnancy, whether it was planned or unexpected, and their future expectations about their life; (2) their knowledge of family planning methods, the barriers in using them and the changes that they believed are necessary to provide a better future for the next generations; (3) the obstacles they faced in obtaining schooling and education; (4) the obstetric care that they had.

V.E.T. led the interview in English. M.S.A.B. (second author) and M.S.T. (third author), who are native speakers of Swahili and fluent in English, interpreted the questions in Swahili and the answers back to English. All interviews were audio recorded. 

At the end of the interview, detailed counselling about family planning and empowering the young mothers about their future development in both personal and professional aspects was performed by the interviewers. 

The recording was transcribed verbatim by V.E.T., M.S.A.B. and M.S.T. confirmed that the English transcription corresponded accurately with the Swahili answers in the recordings. The participants are described with case numbers to preserve anonymity.

Two investigators, V.E.T. and L.M. (seventh author), examined the transcripts independently for key domains to identify emerging themes. Then, they created a master codebook with phrases and words correlating to the themes, so that coding was consistent across interviews. They uploaded the transcripts on the free online platform Taguette ® to facilitate highlighting relevant points and sorting them into key themes according to the codebook. A third investigator, F.M. (sixth author), reviewed the work to ensure codes were correctly applied. V.E.T. and L.M. compared their findings and discussed any discrepancies to reach a consensus on their results. 

## Results

Twelve girls were approached during the study period, and all agreed to participate and were enrolled in the study. Their median age was 18 years old (y.o.) (range 15-18 y.o.). Eight of the girls had attended secondary school, with only one passing her final exams. One girl had never attended school and three other girls had only attended primary school. All were primigravidae and had never used any method of contraception. They were generally healthy with negative HIV status. 

Table 1 displays detailed participant characteristics. 

**Table 1:** Demographic details and general health and obstetric history of the interviewed girls in MMH of Zanzibar, 2022. a: It is presented in years. b: Low-income family: Monthly family income < 300.000 shillings; middle-income family: monthly family income = 700.000-1.000.000 shillings; high-income family: monthly family income > 1.000.000 shillings

Participants	Age^a^	Relationship status	Living arrangements	Education level	Work	Family income^b^	Age of 1^st ^sexual intercourse^a^	Number of sexual partners
Case 1	18	Married	With husband	Secondary	Nursery assistant	Low	17	1
Case 2	17	Single	With parents	Primary	Housekeeping	High	16	1
Case 3	17	Fiancé	With parents	Secondary	Housekeeping	Low	17	1
Case 4	17	Married	With husband	Technical	Never	Middle	17	1
Case 5	17	Married	With husband	Primary	Agricultural work	Low	16	2
Case 6	15	Single	With parents	Secondary	Never	Low	14	1
Case 7	18	In a relationship	With parents	Secondary	Never	Low	17	1
Case 8	18	Married	With husband	Secondary	Never	Middle	18	1
Case 9	18	Married	With husband	Secondary	Never	Low	16	1
Case 10	18	Married	With husband	Secondary	Never	Low	17	1
Case 11	18	In a relationship	With sister	No school	Sales assistant	Low	16	3
Case 12	18	Single	With sister	Primary	Housekeeping	Low	17	1

The main themes that emerged from the interview data were the effects of pregnancy on their life, lack of family planning, obstacles to schooling and education, and non-compliance with obstetric care, which are discussed below. 

Life effects of pregnancy

Pregnancy has had an important impact on the lives of young girls. Most of them mentioned that the pregnancy affected their emotions negatively, particularly as they had expected to continue their education, rather than becoming mothers so soon. 

Half of the young mothers said that they felt “bad” when they found out that they were pregnant because they didn’t plan to become mothers. However, three said that they felt this was “normal”, even though they hadn’t planned it.

Case 2, 18 y.o.: “I feel bad. I am very young for this pregnancy.”

Case 12, 18 y.o.: “I felt normal, but I didn’t expect to be pregnant.”

When we asked “Some years ago, how did you imagine yourself at your current age?”, 10 of them responded that they had hoped to carry on with their studies. One girl hoped to study arts, while others wanted to become nurses, teachers or businesswomen.

However, nine of them presented a positive message for their future and they said that they planned to continue their education when their child becomes a little older. Finally, they emphasized that they believed that they were going to succeed to make their dreams come true. 

Case 6, 15 y.o.: “I will continue school. I will leave my baby with my mum (auntie). I think I will succeed being a doctor in the future and pursue my dream.”

Case 12, 18 y.o.: “I believe that I am goanna succeed to be a mama and a business-woman and to overcome all the difficulties.”

Insufficient family planning 

The pregnancies were mostly unplanned, making the girls feel unprepared for motherhood.

In fact, none of the girls had used any form of contraception and eleven out of twelve hadn’t planned to become mothers at this point of their life. 

For example, when Case 7 (18 y.o.) was asked about the use of any contraceptive method, despite persistent prompting, she repeatedly answered that she did not want to use contraception, without explaining why.

On the other hand, 10 girls mentioned insufficient knowledge and “very few lessons” at school about contraceptive methods. They often shared that they didn't recall most of the information provided at school.

Case 5, 17 y.o.: “I don’t know what contraception is.”

Case 1, 18 y.o.: “I had some lessons in school, but I have forgotten what the teachers told me about this subject.”

Moreover, they referred to discussions with their family and friends, counselling them not to become pregnant at a young age, without mentioning ways to prevent pregnancy.

Case 4, 17 y.o.: “Always they were telling us not to be pregnant, but nobody explained us how.”

A concerning finding was that four of the girls who were interviewed considered discussion about contraception culturally inappropriate.

Case 10, 18 y.o.: “I don’t want teachers to talk about these things in school, they gonna increase sex rates in young age.”

Case 9 ,18 y.o.: “No I don’t think is good because we are young girls those things can not be said at school.”

Their opinion however changed during the discussion. All agreed in the end that having more information on the subject would be beneficial and stated that they would consider using contraception in the future. 

Case 10, 18 y.o.- at the end of the interview: “Yes. I will use contraception in the future. I don’t know which method; I will consult the doctors. After our discussion, I want family planning to be a lesson in schools.”

Ten of the participants wished they had had better education about family planning. They suggested that the best source for providing this information are doctors, as opposed to teachers or family members. 

Case 2 ,17 y.o.: “I would like to have more information about contraception and family planning so as the girls to be able first to fulfil their dreams and then to start their families.”

Case 12 ,18 y.o.: “I believe, doctors should do something in order to improve our knowledge about family planning, maybe through media.”

Case 9 ,18 y.o.: “Finally, I believe that lessons about family planning may help. I may use contraception in the future, I will discuss it with my husband.”

Abortion was something that frightened most of the girls due to religious and cultural beliefs and fear of possible risks that they had heard from relatives. 

Only two girls thought about having an abortion when they found out they were pregnant, although 11 out of 12 participants had had an unwanted pregnancy. The reasons for not considering having an abortion were mainly that they believed that their pregnancy was God’s will. They also had heard from family members fearful stories about fatal bleeding after an abortion, due to “God’s anger”. Some of the more interesting answers that we received were: 

Case 2, 18 y.o.: “No. It’s a God’s will that I am pregnant, and I should raise that kid.”

Case 11, 18 y.o.: “No. My grandma used to tell me not to have any boyfriend and if I have a boyfriend and become pregnant, I should never make an abortion because I will die.”

Case 9, 18 y.o.: “No, I believe when you abort you die. You bleed a lot.”

Case 6, 15 y.o.: “No, because I was afraid of God’s anger.”

Obstacles in schooling and education 

Superstition, religion, difficulties coping with a demanding educational system and financial constraints affected many life decisions, including education and continuation of the pregnancy. Some girls were taken away from school by their families before their pregnancies, because of superstitions surrounding malaise, pain, anger, failed exams or financial problems.

Three girls said that their parents took them away from school because they had an episode of fainting or a panic attack during school hours and they were told that “Satan” or “demons” were inside them. Their parents referred them to witch doctors or religious leaders to help them. 

Case 2, 18 y.o.: “I stopped school because one day I had a panic attack and my parents believed that Satan was inside me. I believed this too. They took me to witch doctors and now I am cured.”

Case 5, 17 y.o.: “I stopped school because demons were inside me, I was beating people during my crisis. I went to the church and now I am cured.”

Case 10, 18 y.o.: “I didn’t take all the exams because I had an episode of fainting. I had spiritual issues. I went to religious doctors. They didn’t succeed to treat me. Then I came to the hospital.”

Moreover, four of the girls referred to the difficulty of the school exams, and their failure to access secondary school or college.

Case 8, 18 y.o.: “I went up to the final class of secondary school, but I failed on the final exams. I was imagining myself still studying but because I don’t have the proper intellectual level is difficult for me. I wanted to be a teacher.”

Case 12, 18 y.o.: “I didn’t pass the exams from primary to secondary school, and I didn’t want to take the exams again.”

Finally, another four of the girls said that they wanted to continue their education, but they could not do so, due to financial difficulties. 

Case 3, 17 y.o.: “I wanted to study to become a tailor, but my family couldn’t support me due to low income.”

Case 11, 18 y.o.: “I never thought of going back to school because I had many financial problems.”

These girls did not return to secondary education; instead, they remained outside school and unemployed, stating that they lacked job opportunities and had no personal goals. It was during that time that they became pregnant. 

Noncompliance with standard obstetric care 

Most of the girls did not have the standard recommended obstetric care, despite the advice of the local doctors.

Eight out of the twelve girls started their obstetric care in the second trimester, despite knowing they were pregnant a lot earlier and although they were aware that obstetric care starts within the first trimester. They stated that they had other priorities rather than their pregnancy. 

Case 1, 18 y.o.: “I understood that I am pregnant during 1st trimester, but I couldn’t go to the hospital because my husband had an accident at this time, and I was taking care of him.”

Case 4, 17 y.o.: “I started attending clinic at second trimester. My sister wanted me first to be married and then to attend the clinic because she was afraid how they goanna face me as a non-married pregnant young girl.”

Case 6, 15 y.o.: “I went for obstetrical screening at the second trimester because during the first trimester I was busy with police issues in order to prove that I was not abused since I am 15.” 

Two of the girls found out that they were pregnant at the second trimester, due to previous menstrual irregularities. 

Still, 11 of them said that they were satisfied with their obstetrical care, although clinics were far from their homes. 

Case 2, 17 y.o.: “I didn’t face any major problems in my obstetric care, although I had only one small hospital near my house.”

## Discussion

The 12 adolescent girls who were interviewed in this study described their pregnancy as a life-changing event that interfered negatively with their future education and career prospects. Although some of the girls left school because of their pregnancy, others had left well before conceiving, because of a variety of superstitious or religious beliefs, and educational or financial difficulties.

Adolescent pregnancy is common in Tanzania, where two out of five girls get married before 18 years and thousands drop out of school due to pregnancy [6]. For many children in Tanzania, education ends after primary school, and access to schooling is much worse for girls, given that Tanzanian society is patriarchal [6]. The 2010 Tanzanian Demographic and Health Survey showed that only 1.7% of girls continued their education in secondary schools, with numbers being even smaller for those living in rural areas [6]. After stopping school, the young girls in our study stated that they lacked job opportunities and had no personal goals. It was during this time that some of them first engaged in unprotected sexual intercourse and became pregnant. As a reflection of higher dropout rates from school, teenagers in rural areas, are considerably more likely to become pregnant, with rates reaching 45% in the Katavi and 43% in the Tabora regions [7]. 

Since 2000, great efforts have been made in Africa to provide incentives to remain in education, such as providing alternatives for those failing their exams or help for those with limited financial resources. A positive step forward was that three sub-Saharan countries, including Tanzania, repealed laws banning adolescent mothers from returning to public schools [1]. According to the 2021 legislation, adolescent mothers in Tanzania are allowed to return to public schools, the latter being required by law to accommodate them [1].

Even before this new legislation was enacted, there was obvious progress in the number of children enrolled in African schools, with the rise being more impressive among primary school children. The proportion of children under 12 years of age who are not in school in Africa halved - from 35% in 2000 to 17% in 2019 [8]. During the same period, the number of children staying away from upper secondary school went down from 63% to 53% [8]. On the other hand, in Tanzania, the dropout rate remains alarmingly high; for primary and secondary school students, the dropout rate is 81% and 35% respectively [6].

However, despite continent-wide progress, the education rate of young Africans remains very low. In 2019, there were approximately 105 million children of primary and secondary school age who were out of school in Africa [8]. More specifically in Tanzania, it is estimated that a total of 5.1 million children aged 7 to 17 are out of school, according to a 2017 report by Human Rights Watch.

Africa is a continent with an impressive proportion of a young and active population [8]. Currently, 61% of the population in Tanzania is under the age of 35 and 60% of Africans are under the age of 25; by 2050 they will represent half of the African population [8, 9]. Africa and more specifically Tanzania have a great potential to leverage their human capital and become a powerful continent by educating their young people and creating skilled and empowered adults [8, 9]. Education has been proven throughout history, to inspire people and is associated with more peaceful communities, greater civic engagement, and stronger democracies [8]. 

Another interesting finding of our study was the fact that many of the girls showed increased trust in local healers and religious leaders, so much so, that they preferred to resolve their health problems with local ‘so-called doctors’ rather than with organized health services within hospitals. This is common practice in Tanzania, where religion plays an important role in everyday life and the opinion of spiritual leaders is highly respected. Traditional medicine has been recognized by the WHO as “respected practices that aim at maintaining health and preventing physical and mental illness” [10]. In an ideal world, Western and traditional medicine should work in cooperation for the benefit of the patient with traditional healers and religious leaders helping to spread important health information, including family planning. A recent qualitative study from Tanzania showed that women, in particular, favoured family planning education by a religious leader, although they preferred for this to take place in a clinic versus a church or mosque [11].

Increased contraception uptake can be further reinforced through having open discussions about family planning and sexual health, both within schools and within society. Steps are certainly being taken towards this direction in Tanzania, as shown by a significant rise in contraception usage, from 23% in 2004 to 34.3% in 2016 [3]. This improvement is attributed to increased partner education levels and home visits by family planning workers [12]. The Zanzibar Ministry of Health (MoH) approved an annual action plan in June 2019 to increase access to long-acting and permanent contraceptive methods (LAPMs) in nine hospitals on the Unguja and Pemba islands. The plan aims to accelerate family planning uptake in support of the implementation of Zanzibar’s Costed Implementation Plan 2017-2022 [13]. 

Despite these remarkable efforts, this was not reflected in teenage childbearing rates, rating from 23% in 2010 to 27% in 2016 and stabilizing at 22% recently [3, 7]. Moreover, Zanzibar has the lowest modern contraceptive prevalence rate of all the zones in Tanzania [12]. Only 27% of sexually active women aged 15 to 49, who need family planning use modern methods of contraception, compared to about half of sexually active women in mainland Tanzania [13].

Barriers to contraceptive method uptake include fears of side effects, misconceived rumours regarding contraceptive methods, usage of birth control of doubtful efficacy, lack of male involvement, and poor access to long-acting reversible contraception. such as the Intrauterine Contraceptive Devices (IUCD) [14-15]. Therefore there is a need to improve Tanzanians' use of modern contraceptive methods, as well as increasing accessibility to long-acting and permanent contraception [14-15]. 

Termination of pregnancy was not considered an option for the interviewed girls. Instead, they mentioned fear of abortion, stating horrific stories about bleeding and death. Abortion is legal in mainland Tanzania when performed for the preservation of the life or mental and physical health of the pregnant woman, as well as in cases of sexual violence [16]. However, unclear abortion policies, leave many women confused about their rights, leading them to undergo unsafe abortions, to such a degree that the latter contribute to 30% of maternal deaths [16].

In this study, we were able to provide a snapshot of how the lives of young girls were affected by an unplanned, precocious pregnancy. Despite important insights about their views and attitudes, a limitation to this study, is the relatively small number of participants and the fact that it took place in the main hospital of Zanzibar. Moreover, Zanzibar is a semi-autonomous province of islands of Tanzania with a population of 1.8 million [17]. Historically, it was habited and influenced by Africans, Arabs, Europeans and Indians. Thus this sample may not be representative of the whole Tanzanian population [17].

A positive finding of this study was that all 12 girls were eager to participate in the study, were open to discussion, and appeared to embrace views regarding family planning that they had not previously considered. Conservative views regarding contraception and sexual education appeared to be dispelled and all girls seemed to appreciate the importance of receiving accurate information.

## Conclusions

Adolescent pregnancy remains an important public health issue in Tanzania, despite important measures by authorities to curtail it. More educational opportunities, financial support, better reproductive health education, and removing cultural and superstition barriers in society may help to reduce adolescent pregnancy rates and retain young girls in schooling. Time is necessary for those changes to show results but with proper planning, future generations may increase their uptake of modern contraceptive methods and delaymotherhood into adulthood.

## Appendices

Appendix 1 

Interview Guide

Questionnaire

Thank you for taking the time to complete this questionnaire. 

A. Demographics 

1. Name:

2. Age: 

3. Which one of the statements below best describes your living arrangements? 

Living alone 

Living with a partner 

Living with a parent/s  

Other 

If “other”, please specify:

4. If you have children, please state how many:

5. Their ages:

6. Which one of the statements below best describes your personal responsibilities regarding dependent children? 

I am not a carer for any dependent children

I am the prime carer of a dependent child/children 

I am a carer of a dependent child/children but someone else is the prime carer 

I equally share the care of a dependent child/children 

Other  

If “other”, please specify: 

7. Which is your relationship status?

Married

Separated 

In a relationship

Single 

B. Education 

8. Please indicate the highest level of education you have achieved: 

Primary

Secondary

Technical

9. Please indicate the highest level of education your mother has achieved: 

Primary

Secondary

Technical

Tertiary

10. Please indicate the highest level of education your father has achieved: 

Primary

Secondary

Technical

Tertiary

C. Employment Details 

11. Do you work?

Full time                          

Part-time

Householding 

Full-time education

Education/Working 

12. How long do you work? 

Less than 1 year 

1-3years

4-6years

Please specify the number of years: ____ 

D. Living conditions 

13. Which is the average monthly income of your parents in case that you live with them/of you in case that you live alone?

300.000 shillings

700.000-1.000.000 shillings

> 1.000.000 shillings

E. Medical Information 

14. Did you have the mandatory vaccination when you were born?

Yes 

No

Other 

15. How many visits did you attend to pediatrician as a kid for routine examination?

1-3

4-10

>10

F. Gynecological history 

16. Age of first sexual intercourse:

17. Number of sexual patterners:

18. HIV status: 

Interview

Do you have any questions before we start?

1.     Can you remember how you felt when you found out you were pregnant? 

2.     A couple of years ago, how were you seeing yourself at your age? 

3.     Do you feel this pregnancy is making major changes to your life? In what way? 

4.     Looking back on the years before, did you think that you had adequate information about contraception? 

5.     Who provided you with this information? 

6.     Did you feel that this information helped you?

7.     Would you have preferred this information to be given in a different way?

8.     How easy was to find contraception (condoms, pills etc.)?

9.     Did you ever think about having an abortion? 

10.  Will you use contraception in the future? 

11.  When you understand that you were pregnant, did you go immediately to the obstetrician? When was your first obstetrical visit? (1st,2nd, 3rd trimester?)

12.  Do you attend the routine obstetrical screening during this pregnancy? Was it easy for you? What are the barriers that you face concerning your obstetrical care?  

13.  Who do you think going to support you raising this child? In what ways? 

14.  How do you imagine your life in the future? 

15.  What do you expect to change in the future for young girls in your country to have a better support on these subjects?

Thank you for taking the time to complete this questionnaire.

Appendix 2

Informed Consent

Researcher: Triantafyllidi Varvara Ermioni

Title of Project: Qualitative analysis of adolescent pregnancy in Tanzania, Zanzibar through a semi-structured interview.

I am asking for your voluntary participation in my science fair project by accepting to be interviewed. Please read the following information about the project. If you would like to participate, please sign in the appropriate area below. 

Purpose of the project: We aim to explore the educational, healthcare, and socioeconomic barriers pregnant adolescents may face in Zanzibar, Tanzania always with respect to the patients with a major further goal to be a part of the improvement of obstetrical and gynaecological healthcare that is provided to adolescent women as well as to support their education and their social development.

Time required for participation: 30-40 min

How confidentiality will be maintained: The study will not contain any names of the participants. The names are only asked for the reason of authentication of the records of this study.

If you have any questions about this study, feel free to ask directly the researcher before the interview. 

Voluntary Participation: 

Phone/email: ____________________ 

Participation in this study is completely voluntary. If you decide not to participate there will not be any negative consequences. Please be aware that if you decide to participate, you may stop participating at any time and you may decide not to answer any specific question. 

By signing this form I am attesting that I have read and understand the information above and I freely give my consent/assent to participate or permission for my child to participate. 

Adolescent Informed Consent 

Parental/Guardian Permission 

International Rules: Guidelines for Science and Engineering Fairs 2016 -2017 (student.societyforscience.org/intel-isef (Page 37)) 
